# Formulation Strategies for Folate-Targeted Liposomes and Their Biomedical Applications

**DOI:** 10.3390/pharmaceutics11080381

**Published:** 2019-08-02

**Authors:** Parveen Kumar, Peipei Huo, Bo Liu

**Affiliations:** Laboratory of Functional Molecules and Materials, School of Physics and Optoelectronic Engineering, Shandong University of Technology, Xincun West Road 266, Zibo 255000, China

**Keywords:** folic acid, liposomes, folate-targeted, phospholipids, rheumatoid arthritis

## Abstract

The folate receptor (FR) is a tumor-associated antigen that can bind with folic acid (FA) and its conjugates with high affinity and ingests the bound molecules inside the cell via the endocytic mechanism. A wide variety of payloads can be delivered to FR-overexpressed cells using folate as the ligand, ranging from small drug molecules to large DNA-containing macromolecules. A broad range of folate attached liposomes have been proven to be highly effective as the targeted delivery system. For the rational design of folate-targeted liposomes, an intense conceptual understanding combining chemical and biomedical points of view is necessary because of the interdisciplinary nature of the field. The fabrication of the folate-conjugated liposomes basically involves the attachment of FA with phospholipids, cholesterol or peptides before liposomal formulation. The present review aims to provide detailed information about the design and fabrication of folate-conjugated liposomes using FA attached uncleavable/cleavable phospholipids, cholesterol or peptides. Advances in the area of folate-targeted liposomes and their biomedical applications have also been discussed.

## 1. Introduction

Cell proliferation and survival are dependent on the ability of the cells to obtain vitamins. Eukaryotic cells require folic acid (FA) and its reduced counterparts for carbon transfer reactions for the biosynthesis of the nucleotide bases. However, the passive membrane permeability of FA is minimal at physiological pH and temperature due to the presence of two terminal carboxylate moieties. To alleviate this obstacle, two mechanisms have been evolved by nature for their cellular internalization. One mechanism is facilitated by the low membrane-spanning proteins (K_D_ ~ 1–5 µM), which directly transport reduced folate counterparts into the cell cytosol [1]. In another mechanism, a high-affinity glycoprotein receptor (K_D_ ~ 100 pM), which is generally referred to as folate receptor (FR), preferentially mediates the uptake of folate in oxidized form into the cells via endocytosis [2]. FR has four known isoforms, i.e., FR-α, FR-β, FR-γ, and FR-δ, which are differentially expressed in individual tissues [3]. FR-α, FR-β, and FR-δ are glycosyl phosphatidylinositol-anchored membrane proteins [4,5,6,7], while FR-γ is constitutively secreted by the lymphoid cells [8]. FR-α is overexpressed in more than 90% of ovarian carcinomas, and at an even higher frequency in the other epithelial cancers; FR-β is overexpressed in activated macrophages [9,10] and myeloid leukemia [11]; FR-δ is found to be expressed in regulatory T cells [12]. FRs show high affinity for FA, i.e., *K*_D_ ~ 0.1 nM (FR-α) [13], *K*_D_ ~ 1 nM (FR-β) [14], and *K*_D_ ~ 0.4 nM (FR-γ) [7]. However, while the reduced folate carriers are ubiquitously expressed, their affinity (K_D_) is in the µM range, and FRs have a more than 10^3^-fold higher affinity for folate, which enables their in vivo targeting via folate conjugation without any concerns of the potential interference from the binding of much weaker reduced folate carriers. Moreover, FRs have also been found at significant levels in some normal epithelia, which are involved in the retention and uptake of folate, primarily in the placenta, intestine, lungs, kidney, and choroid plexus [15,16,17]. Since these FRs are localized on apical surfaces of the polarized epithelia, they are usually inaccessible to the blood-borne folate-conjugates. Therefore, the expression of tumor tissue-selective FRs and the lack of expression of the accessible FRs in normal tissue suggest them as a selective marker for the targeted delivery of therapeutics to disease cells. FR can effectively transport FA and folate-conjugated cargos including therapeutics, proteins, imaging agents, liposomes, nanogels, etc. In particular, folate-targeted liposomes have been widely used to deliver a variety of compounds to cancer cells, including genes [18,19,20,21,22], chemotherapeutic agents [23,24,25,26,27], imaging agents [28,29,30], and antisense oligonucleotides [31,32].

**Figure 1:**
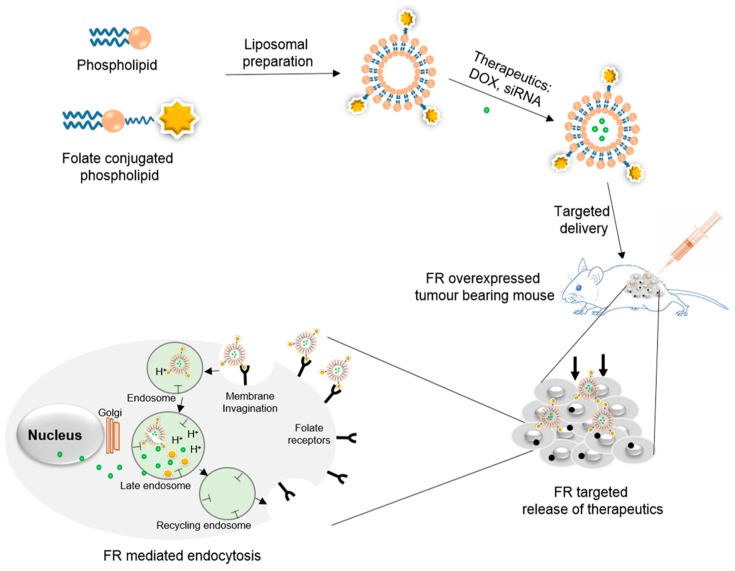
Schematic representation exemplifying the preparation of folate-conjugated liposomes and targeted delivery of therapeutics via FR-mediated endocytosis (exogenously added folate-conjugated liposomes binding specifically to FR proteins with high affinity. The plasma membrane invagination occurs around liposomes/FR complex to form endosomes (intracellular vesicle). The receptor changes conformation to release the conjugate after acidification (~pH 5) of the lumen of maturing endosomes. Eventually, the fates of the liposomal components, encapsulated therapeutics, and FRs are determined within late endosomal elements during the sorting process).

Liposomes are non-toxic, biocompatible, and biodegradable, and can help to increase the efficacy, provide stability, and reduce the toxicity of the encapsulated drugs. Liposomes are phospholipid vesicles composed of concentric phospholipid bilayers that enclose discrete aqueous spaces. The phospholipid tails generally consist of two hydrophobic long fatty acid chains and are driven by hydrophobic interactions to form fatty acid tails aggregation to minimize interactions with water molecules. Phospholipids are amphiphilic and have excellent biocompatibility in nature, and are therefore highly suitable to be employed as vital pharmaceutical excipients. Hydrophobic molecules can be inserted into the bilayer membrane, whereas hydrophilic molecules can be entrapped in the aqueous center [33,34,35]. Therefore, liposomes are considered to be model biomembranes, providing impressive efficacy and safety to site-specific drug delivery [36]. Folate-targeted liposomes have been prepared by using FA-conjugated lipids, cholesterol, and proteins, and have been synthesized by reacting carboxylic group of FA to complementarily reactive group of lipids, cholesterol, and proteins. The therapeutic encapsulated liposomes have been delivered to the FR-overexpressed tumors, where they bind with FRs, which are externally oriented on the cell membrane. After binding, the membrane that surrounds the liposome/FR complex immediately starts to invaginate in order to form a distinct internal vesicle (early endosome) within the cell. The FR changes conformation to release the folate-conjugate after the acidification (~pH 5) of the vesicle lumen [37,38]. Eventually, the fates of the liposomal components, encapsulated therapeutics, and FR are determined within the late endosomal elements during the sorting process (Figure 1). The cytosolic entry of the FA can occur at this point by (a) direct membrane translocation of protonated FA species; (b) simple leakage of folate during improper membrane fusion processes [39]; and (c) anion exchange transportation of FA out of the endosome [40].

The basic principle for the fabrication of folate-conjugated liposomal systems is to attach the FA with the liposomes using folate-linked phospholipids, cholesterol or peptides. The design and fabrication of folate-conjugated liposomes require a detailed understanding of biomedical and chemical science. Therefore, the present review aims to provide the detailed information about the advances in the field of folate-targeted liposomes and their biomedical applications, in order to assist the reader to comprehend this emerging field at a single platform. This review summarizes the updated synthetic strategies for the fabrication of folate-conjugated liposomes and their biomedical applications. We propose that a summarized review will prompt novel ideas among researchers in the design and fabrication of new and effective folate-targeted liposomal delivery systems.

## 2. Design and Fabrication of Folate-Conjugated Liposomes

Thin-film hydration is the most frequently adopted conventional method for the preparation of liposomes, in which lipid components with or without therapeutics are dissolved in an organic solvent, followed by the removal of the solvent to form a thin film, further followed by rehydration in water or buffer. Other conventional methods include ethanol injection, reverse-phase evaporation, and freeze-drying [41]. With these conventional methods, liposomes can be formulated and tuned to the required composition, charge, size, and lamellarity. Post-processing techniques such as extrusion, homogenization, sonication, and freeze-thawing are required to control their size and distribution. There are various reports of extrusion techniques for liposome preparation using several devices such as the Nuclepore 24 mm filter holder [42,43], Lipex extruder [44,45], Millipore high-pressure filter holder [46], mini-extruders (Hamilton syringes) [47], Schleicher and Schuell ultrafiltration device [48], high-pressure Plexiglas filtration cells [49], Dispex Maximator extruder [50], and Avestin Liposo-Fast extruder [51]. However, multi-functional liposomes usually require more processing steps, such as conjugating imaging molecules and/or to adding a targeting ligand, which result in poor control over the surface and size properties due to batch-to-batch variations, limiting the in vivo results [52]. These complications in the fabrication of multifunctional liposomes for the targeted delivery restrict the scaling-up of their production, which is one of the major reasons for which no targeted liposome has yet received clinical approval.

Microfluidic technology, such as the microfluidic hydrodynamic flow focusing method, has appeared as a relevant alternative to conventional bulk methods for the fabrication of multifunctional liposomes in single-step [53,54]. In this method, organic solvent-containing lipids introduced from a central microchannel are mixed with an aqueous buffer solution from the vertical channels in a well-controlled manner. The solubility of the lipids decreases as the organic solvent diffuses in the aqueous solvent, and the lipids self-assemble into planer lipid bilayers, which subsequently bend to minimize the exposure to the hydrophilic aqueous buffer in order to finally close into spherical liposomes [55]. This method provides good control over liposomal properties, including surface properties (targeting ligands and PEGylation), particle size, and size distribution [56]. Moreover, the method also enables the combinatorial synthesis of liposomal libraries [57] with systematically variable properties, which could prove to be an effective way to discover novel therapeutics in the pharmaceutical industry via high-throughput screening. Furthermore, the preparation process can easily be scaled-up using parallel integrated microfluidic devices [58,59] or a high aspect ratio, which is convenient for massive industrial production and clinical applications [60,61].

**Figure 2:**
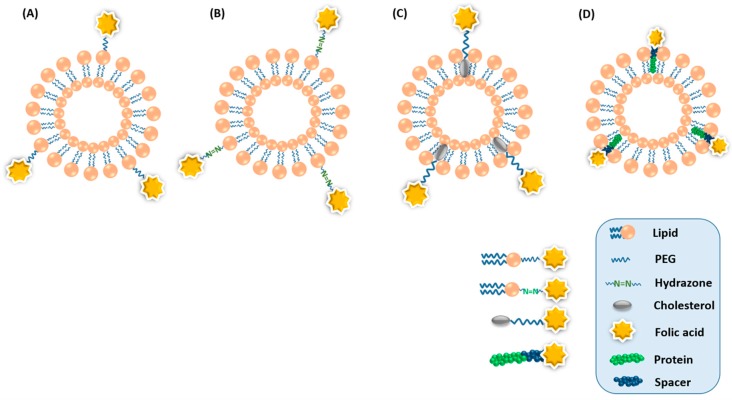
Pictorial representation of folate-conjugated liposomes composed of FA linked with (**A**) phospholipid, (**B**) acid-labile phospholipid, (**C**) cholesterol, and (**D**) protein.

Folate-conjugated liposomes loaded with therapeutics have received much interest due to their enhanced tumor uptake [62,63,64,65]. In recent decades, various approaches have been used for the design and successful fabrication of folate-conjugated liposomes [66,67]. There are two potential approaches for incorporating FA into liposomes. The first approach involves the pre-formation of the liposomes, followed by the conjugation of FA on the surface functional groups. However, this approach has some serious downsides. The conjugation reactions with preformed liposomes cannot be precisely controlled. The reactive agent may diffuse the lipid bilayer and react with head-groups of both sides of the bilayer. Therefore, the inner core may contain by-products, and encapsulated therapeutics may interfere with the by-products and reactive agents. Moreover, reaction conditions such as the use of organic solvents, e.g., methanol and chloroform, could compromise the integrity of the lipid bilayer. The second approach is used to overcome these serious downsides, in which FA is attached to the lipid head groups prior to liposome formation. Since FA is chemically stable in organic solvents, pre-synthesis of FA-conjugated lipid and inclusion during liposomal formulation can be easily accomplished. Moreover, since FA-conjugated lipids dissolve easily in organic solvent, their incorporation into the liposomes is highly feasible during preparation. Furthermore, liposomes with precise compositions can be prepared, because it is possible to obtain chemically purified FA-conjugated lipids. The incorporation of even 0.1 mole% of folate-conjugated lipid during liposome preparation is sufficient for effective targeting to FR-bearing tumor cells [68]. Since liposomes mainly consist of phospholipids, cholesterol, and, in some cases, targeted peptides, FA has been directly conjugated with them before the formation of liposomes. Figure 2 shows a pictorial representation of FA-conjugated liposomes composed of FA linked with phospholipid, acid-labile phospholipid, cholesterol, and protein.

### 2.1. Design and Fabrication of Folate-Conjugated Phospholipids

The majority of FR-targeted liposomes have been synthesized using the addition of folate-conjugated phospholipids during the formation of liposomes. However, Lee et al. [69] showed that folate-conjugated liposomes were not recognized by FR when FA was directly attached to the phospholipids. Instead, receptor binding activity was only restored when FA was attached to the liposome via a long PEG-based spacer. Therefore, FA has been conjugated to the phospholipids via PEG spacer. In addition, PEG modification also provides the benefit of increasing the solubility and prolonging the half-life of the drug in the body, as well as passively assisting in enhancing the drug accumulation at the tumor site [70,71,72,73]. Moreover, FA modification can further enhance the retention of liposomes in the tumor and facilitate its uptake via FR-mediated endocytosis, enabling the drug to target FR-overexpressed tumors more precisely and efficiently [74]. Until now, two types of folate conjugation to the phospholipids have been used: (a) uncleavable folate conjugation [75,76,77,78,79,80], and (b) cleavable folate conjugation [81].

#### 2.1.1. Un-Cleavable Folate-Conjugated Phospholipids

**Figure 3:**
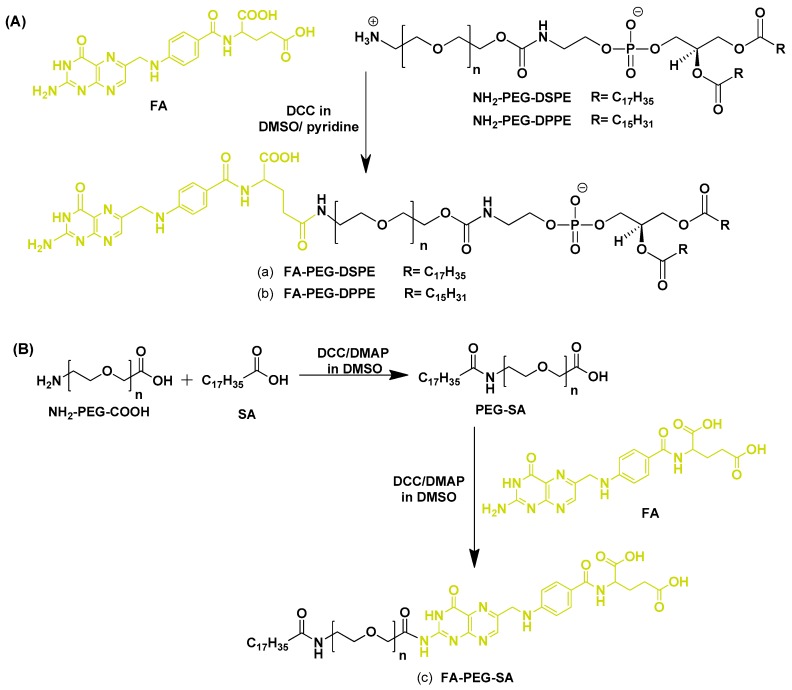
Synthetic scheme for uncleavable FA-conjugated phospholipids. (**A**) (**a**) FA-PEG-DSPE Reproduced with permission from [83]. Copyright 2013, DOVE Medical Press; (**b**) FA-PEG-DPPE. Reproduced with permission from [82]. Copyright 1999, American Chemical Society. (**B**) (**c**) FA-PEG-SA. Reproduced with permission from [84]. Copyright 2018, DOVE Medical Press.

Un-cleavable folate-conjugated phospholipids are widely used for the formation of folate-targeted liposomes. Folic acid-poly(ethylene glycol)-distearoylphosphatidylethanolamine (FA-PEG-DSPE), folic acid-poly(ethylene glycol)-dipalmitoylphosphotidylethanolamine (FA-PEG-DPPE), and folic acid-poly(ethylene glycol)-stearylamine (FA-PEG-SA) are the most commonly used folate-conjugated phospholipids. Lee and Low [68] first reported the synthesis of FA-PEG-DSPE phospholipid for the preparation of folate-conjugated liposomes (Figure 3A). To exploit the FR overexpression in epithelial cancer cells, doxorubicin (DOX)-encapsulated folate-targeted liposomes were fabricated by incorporating 0.1 mol% FA-PEG-DSPE. Folate-targeted liposomal DOX uptake was found to be 45 and 1.6 times higher, whereas cytotoxicity was 86 and 2.7 times higher, when compared with non-targeted liposomal DOX and free DOX with KB cells, respectively. Calcein (fluorescent dye)-encapsulated folate-targeted liposomes almost exclusively internalized by FR-overexpressed HeLa cells from HeLa/WI38 co-culture, indicating their excellent specificity towards FRs.

Gabizon et al. [82] synthesized the FA-PEG-DPPE phospholipid using carbodiimide-mediated coupling of H_2_N-PEG-DSPE to FA (Figure 3A) and formulated liposomes using hydrogenated soybean phosphatidylcholine (HSPC), cholesterol, and 0.5 mol% FA-PEG-DPPE, with or without DSPE-MPEG. FA-PEG-DSPE significantly enhanced the binding of liposomes to tumor cells. Saul et al. [25] also synthesized the FA-PEG-DPPE phospholipid and used this to prepare FR-targeted liposomes to deliver DOX. The folate-conjugated liposomal DOX uptake was dependent on several targeting ligands in folate-overexpressed C6 and KB cells. Preferential uptake in C6 glioma cells relative to cortical cells in C6 glioma/cortical cells co-cultured cells indicated specificity towards FR. Moreover, folate-targeted liposomal DOX exhibited slow proliferation of C6 and KB cells, with negligible effect on the cortical cells. Recently, Gao [79] also utilized FA-PEG-DPPE for the preparation of folate-targeted liposomes for the delivery of DOX, showing selective and specific delivery of DOX to KB cells relative to normal cortical cells.

FA–PEG–SA is another type of folate-conjugated phospholipid widely used for the preparation of folate-targeted liposomes. Gupta et al. [80] reported the fabrication of FA-PEG-SA phospholipid (Figure 3B) to prepare folate-targeted liposomes for enhanced tumor targeting. Liposomes were prepared using HSPC, cholesterol, and FA-PEG-SA. 5-FU containing folate-targeted liposomes exhibited B16F10 melanoma cell binding 11 times higher than conventional liposomes, and in vivo cytotoxicities revealed the IC_50_ of folate-targeted liposomes and conventional liposomes to be 1.87 mM and 4.02 mM, respectively. Administration of 5-FU encapsulated folate-targeted liposomal formulation to B16F10 tumor-bearing Balb/c mice resulted in a significant reduction in tumor growth compared with conventional liposomes and free 5-FU. Recently, Qiu et al. [84] also utilized FA–PEG–SA to synthesize lipid-polymer hybrid nanoparticles with lipid/lipid-PEG shells and polymer cores, which exhibited the characteristics of both polymeric liposomes and nanoparticles. This delivery system was used for the targeted delivery of vincristine, which exhibited remarkable therapeutic effects for lymphoma treatment, and also reduced the systemic toxicity.

#### 2.1.2. Acid-Cleavable Folate-Conjugated Phospholipids

Acid-cleavable folate-conjugated phospholipids have not been much explored for the fabrication of FR-targeted liposomes. Hydrazone bonds are one of the effective acid-cleavable bonds used to couple drugs with targeted antibodies in medical biotechnology. Hydrazone bonds are stable under physiological environment, but rapidly dissociate in acidic environment (such as lysosomes) of the cell. The triggered release of drugs in the acidic environment is beneficial, as this environment is well-correlated with that of cancer tissues, and the application of acid-sensitive liposomes has been proven to be effective in the treatment of cancer [85].

**Figure 4:**
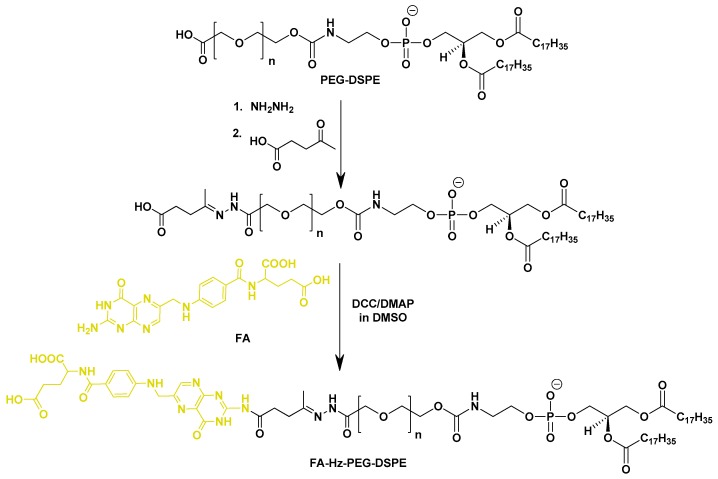
Synthetic scheme for acid-cleavable phospholipid, FA-Hz-PEG-DSPE. Reproduced with permission from [81]. Copyright 2018, Elsevier.

Hydrazone containing linker was first utilized to link monoclonal antibodies with PEG chains for the preparation of liposomes [86] exhibiting improved activity against KLN 205 and HCT-15 cells. Recently, Man Li et al. [81] fabricated a hydrazone-based acid-cleavable phospholipid (FA-Hz-PEG-DSPE) for the fabrication of FR-targeted liposomes (Figure 4). Due to poor tumor cell internalization and poor penetration of the blood–brain barrier, effective therapy for glioma is still lacking, and to overcome this problem, they designed and fabricated paclitaxel (PTX)-loaded liposomes modified with tumor microenvironment-cleavable FA and cell penetrating peptide dNP2 (KIKKVKKKGRKKIKKVKKKGRK-cys). dNP2-modified and FA-cleavable liposomes were prepared by using modified phospholipids, DSPE-PEG-dNP2 and FA-Hz-PEG-DSPE. The size of both non-cleavable (Fd-Lip/PTX) and cleavable (cFd-Lip/PTX) dual modified liposomes was about 104 nm, with a narrow size distribution. The zeta potentials of Fd-Lip/PTX and cFd-Lip/PTX were around −6 mV at pH 7.4, while obvious charge inversion was observed in cFd-Lip/PTX, with a zeta potential of around 6.44 mV, but the zeta potential of Fd-Lip/PTX remained around −6.44 mV at pH 6.8. Furthermore, dNP2 modification significantly enhanced trans-migration across the blood–brain barrier, when tested with in vitro model. Moreover, acid-cleavable dual modified liposomes facilitated the penetration into the cells and enhanced the cytotoxicity of the drug.

### 2.2. Design and Fabrication of Folate-Conjugated Cholesterol Derivative

**Figure 5:**
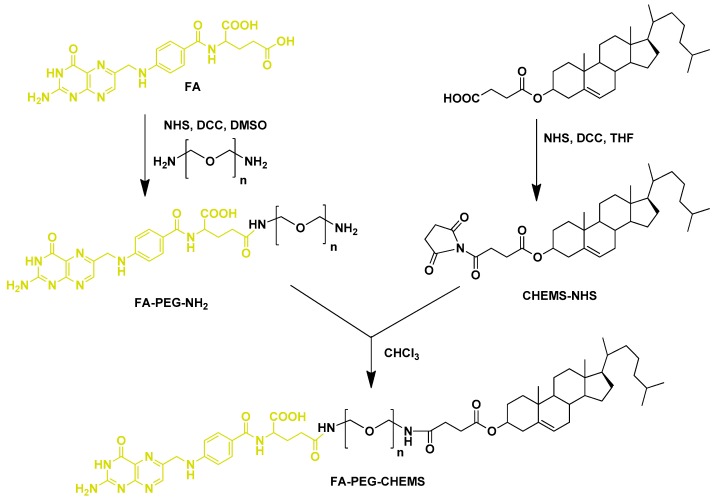
Synthetic scheme for FA-conjugated cholesterol, FA-PEG-CHEMS. Reproduced with permission from [11]. Copyright 2008, Elsevier.

Another feasible and effective mechanism for targeting FR is the conjugation of FA with cholesterol, which is one of the most important constituents for the preparation of liposomes. Since cholesterol provides various benefits, including (a) it can act as an intercalator with phospholipids molecules, (b) it can act as fluidity buffer, (c) it restricts the trans to gauche conformational change, and (d) it also stabilizes the lipid membranes against temperature changes, resulting in low permeability at elevated temperature, thus imparting better stability [87,88,89,90]. CHEMS (3β-hydroxy-5-cholestene-3-hemisuccinate), a derivative of cholesterol, has been widely used with 1,2-dioleoyl-sn-glycero-3-phosphoethanolamine (DOPE) to prepare pH-sensitive liposomes [91,92,93]. CHEMS, a carboxyl-containing weakly acidic amphiphile, stabilizes the DOPE in a pH-dependent manner in bilayer phase. As the pH drops to the level that suppresses the ionization of CHEMS carboxyl, the lipids undergo phase transition with consequent release of liposomal contents. These types of liposomes show great promise as pH-sensitive carriers, as they can maintain good stability in the blood circulation and quickly release the payload in the acidic tumor environment [94,95]. Moreover, conjugation of CHEMS with FA could provide a further added advantage in targeting FR-overexpressed tumors. FA-PEG-CHEMS is one such FA-conjugated cholesterol derivative used for the fabrication of folate-targeted liposomes [96]. Xiang et al. [11] reported the synthesis of FA-PEG-CHEMS (Figure 5) and its utilization as a targeting ligand for liposomal DOX in FR-expressing cells. FA-PEG-CHEMS containing liposomes showed remarkable colloidal stability, and was taken up selectively by FR-overexpressed KB cells. Moreover, these liposomes exhibited enhanced in vitro cytotoxicity (IC_50_ = 10 μM) and longer in vivo circulation (t_1/2_ = 12.34 h) compared with non-targeted liposomes (IC_50_ = 57.5 μM, t_1/2_ = 17.1 h). Chen et al. [97] also used FA-PEG-CHEMS for the preparation of FR-targeted pH-sensitive liposomes co-loaded with DOX and imatinib to overcome multidrug resistance. Folate-conjugated liposomes were prepared by the thin film hydration method with a molar ratio of 40:25:20:20:4:1 of the lipid compositions: DOPE, HSPC, cholesterol, CHEMS, mPEG_2000_-Hz-VES (cleavable D-α-tocopheryl polyethylene glycol succinate (TPGS) analogue), and FA-PEG-CHEMS. The loading efficiencies into liposomes for both of the drugs were about 96% at the 1:8 (w/w) ratio of drug/lipid, with a particle size of around 159 nm. The fabricated folate-targeted liposomes co-loaded with DOX and imatinib significantly enhanced the anti-tumor effect and provided a novel strategy for improving the chemotherapeutic efficacy against multidrug-resistant tumors.

### 2.3. Folate-Conjugated Proteins

Hydrophobic fragments of surfactant protein D (SP-D) linked with FA display a highly effective and innovative strategy for targeting liposomes to FRs. Mammalian pulmonary surfactant proteins can promote self-assembly of phospholipids toward a zero potential interface, indicating that models or fractions of these proteins are well capable of recognizing and interacting with the phospholipids of liposomes [98]. Also, the α-helical neck section of the SP-D exhibits an affinity for phospholipids, which might stimulate the binding of SP-D with the phospholipids of liposomes [99], thereby further supporting the potential use of SP-D as an anchor and linker for FA conjugation.

**Figure 6:**
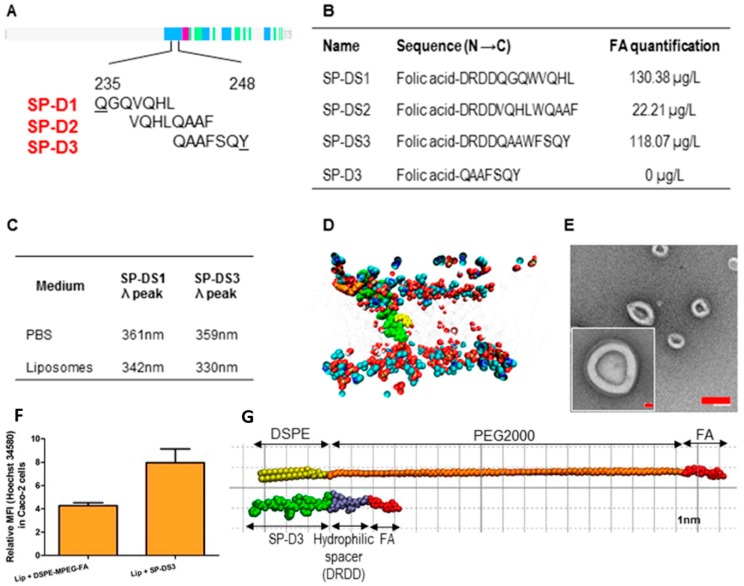
(**A**) Analyzed SP-D fragments for SPD1, SP-D2, and SP-D3. Numbers correspond to the amino acid positions in the SP-D sequence. (**B**) Designed peptide linkers and FA quantification at the liposomal surface. (**C**) Tryptophan fluorescence results of the tested peptides. (**D**) Molecular dynamics simulation of the interaction of liposomal membrane with SP-DS3. FA (orange) at the surface of the membrane and tryptophan residue (yellow) in an apolar environment. (**E**) TEM images of liposomes. Scale bar (red) represents 200 nm at low magnification and 20 nm at high magnification. (**F**) Efficient membrane disruption of SP-DS3 peptide incorporating liposomes compared to FA-PEG-DSPE incorporating liposomes in Caco-2 cells. (**G**) Structures of SP-DS3 and FA-PEG-DSPE. Reprinted with permission from [100]. Copyright 2015, American Chemical Society.

Eugenia Nogueira et al. [100] utilized the bifunctional SP-D peptide-containing neck-domain, which acted as an anchor and a linker for FA to the liposomes. Neck-domain peptides constructed from SP-D were fabricated by covalent conjugation of FA with the N-terminal of peptides via pteroic acid and glutamic acid using an Fmoc-based solid-phase synthetic procedure [101,102], and C-terminal was covalently bound with Oregon Green 488 [103,104]. The peptide was deeply inserted into the lipid bilayer of the liposomes without affecting their integrity. The interaction of the three different peptide sequences (SP-D1, SP-D2, and SP-D3) was analyzed (corresponding to amino acids 235−248 of SP-D) with a liposomal formulation consisting of DOPE, cholesterol, and DSPE-MPEG (Figure 6A). A hydrophilic bi-functional spacer (DRDD) was introduced at the N-terminal to improve the aqueous solubility of the final FA-conjugate peptides (SP-DS1, SP-DS2, and SP-DS3). FA quantification at the surface of the liposomes demonstrated the utility of the DRDD spacer, since the liposomes (SP-D3) without DRDD did not exhibit quantifiable FA (Figure 6B). The tryptophan fluorescence results exhibited a blue shift in the liposomal formulation using SP-D1 (342 nm) and SP-D3 (330 nm), which demonstrated that the SP-D3 peptide penetrated more deeply into the liposomal bilayer than SP-D1 (Figure 6C). Deep insertion of peptides presents the advantage of preventing its dissociation from the liposomes after intravenous injection. Molecular modeling study revealed that FA was located at the surface of the membrane, whereas SP-DS3 was inserted deeply into the bilayer of membrane (Figure 6D). The TEM images showed the spherical morphology resulting from integrating SP-DS3 nanocapsules of liposomes with a uniform diameter (~120 nm) (Figure 6E). Around a 2-fold Hoechst-associated fluorescence was shown by SP-DS3-incorporating targeted liposomes compared with liposomes prepared with DSPE-MPEG in Caco-2 cells (Figure 6F). They proposed that the liposomal membrane disruption mechanism was more effective because of the small size of the hydrophilic bifunctional peptide (DRDD) spacer compared with PEG (Figure 6G). Furthermore, other improved characteristics of these liposomes, such as their lack of cytotoxicity, small size, and specific targeting to FR-overexpressed cells, greatly support their use as a specific drug delivery system for FR targeting.

## 3. Biomedical Applications of Folate-Conjugated Liposomes

A number of therapeutics, including paclitaxel (PTX) [105,106], doxorubicin (DOX) [107,108], methotrexate [109,110], cis-platin [111,112], curcumin [113,114], 5-fluorouracil [115,116], irinotecan [117,118], and nucleic acids [119,120], have been delivered via polymeric carriers [121] to cells. However, liposomes provide unique properties, as their charge, size, and surface properties can easily be tuned by selecting different ingredients of lipid mixture prior to the preparation of the liposomes. Moreover, further functionalization of liposomes with site-specific ligands such as FA possesses the added advantage of targeted delivery, and has also proven to be an effective option for biomedical applications [122,123,124,125,126,127]. In this section, applications of folate-conjugated liposomes for the delivery of various small hydrophilic/hydrophobic anticancer drug molecules and large macromolecules such as DNA and siRNA are discussed. Also, the applicability of folate-conjugated liposomes in inflammatory diseases such as rheumatoid arthritis is also discussed. An overview of folate-targeted liposomal delivery systems has been summarized in Table 1.

### 3.1. Folate-Targeted Liposomes for Anticancer Drug Delivery (Small Hydrophilic/Hydrophobic Molecules)

Small hydrophilic/hydrophobic chemotherapeutics are non-immunogenic compared to other protein toxins, thereby allowing continuous dosing and improved tissue penetration [128]. However, they suffer from several downsides, such as degradation before reaching to the target, reduced therapeutic effect, development of drug resistance, reduced immunity, and serious side effects during and after the treatment. In an effort to overcome these downsides, a variety of nanoformulations have been developed in recent decades. However, the conception of the liposome-based delivery system has made a valuable impact on pharmaceutical research. Because of extensive developments in liposome technology, a variety of liposomal formulations are available on the market for human use, and many formulations are undergoing clinical trials. Moreover, folate-targeted liposomes play diverse roles and hold great potential for targeted delivery of small anticancer drug molecules in FR-overexpressed cells. Folate-targeted liposomes effectively enhance the therapeutic efficacy of various small hydrophilic/hydrophobic anticancer drugs. To evaluate the combined antiangiogenic and cytotoxic properties of anticancer drugs, Peres-Filho et al. [129] developed a paclitaxel and imatinib co-encapsulated liposomal formulation targeted to FR. Conventional liposomes were composed of phosphatidylcholine (PC), cholesterol, and mPEG-DSPE. Folate-conjugated liposomes were prepared by post-insertion of FA-PEG-DSPE and possess a size of about 122 nm, exhibiting significantly higher cell death on MCF7 and PC-3 cells compared with conventional liposomes. Moreover, reduced VEGF gene expression was observed with targeted liposomes in MCF7 and PC-3 cells compared with conventional liposomes. These multifunctional folate-targeted liposomes with multi-drug co-encapsulation suggest multiple attractive antitumor strategies for enhancing drug internalization and accumulation in the targeted cells.

5-Fluorouracil (5-FU)-loaded folate-targeted liposomes were developed by Handali et al. [130] in order to enhance the safety and efficacy of 5-FU. The folate-targeted liposomes were prepared by the thin film hydration method using dipalmitoylphosphatidylcholine (DPPC), cholesterol, and FA-PEG-DSPE. The encapsulation efficiency of the optimized liposomal formulation was about 39%, with a particle size of around 174 nm and a spherical morphology. Differential scanning calorimetry results revealed the amorphous state of 5-FU in the liposomes. In vitro cytotoxicity of the prepared formulation was evaluated against Caco-2, HT-29, CT26, MCF-7, and HeLa cell lines using MTT assay and results showed that the folate-targeted liposomes exhibited higher cytotoxicity than conventional liposomes and free 5-FU. In vivo results demonstrated that targeted liposomal formulation reduced tumor volume (169.00 mm^3^) significantly in comparison with free 5-FU (326.40 mm^3^). Thus, it can be concluded that FA-targeted liposomes may provide an interesting platform for delivery of 5-FU to cancer cells.

**Figure 7:**
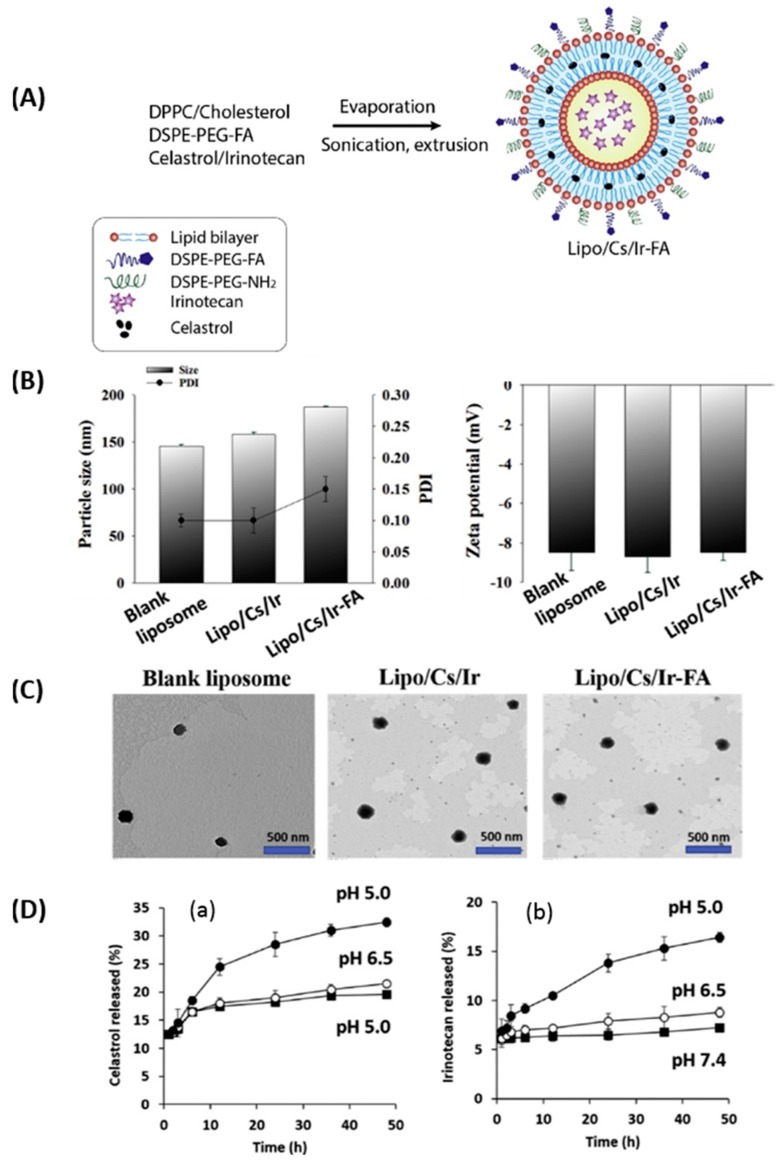
(**A**) Schematic illustration for preparation of FR-targeted liposomes containing drugs (celastrol and irinotecan). Comparison of (**B**) particle size, PDI, and zeta potential. (**C**) TEM images of blank liposomes, Lipo/Cs/Ir, and Lipo/Cs/Ir-FA. (**D**) In vitro release profiles of irinotecan and celastrol from Lipo/Cs/Ir-FA in (**a**) ABS and (**b**) PBS. Reprinted with permission from [122]. Copyright 2018, Elsevier.

Since liposomes can incorporate both hydrophobic and hydrophilic drugs for combined chemotherapy treatment, Soe et al. [122] investigated FA-conjugated liposomes incorporating celastrol (Cs) (hydrophobic) and irinotecan (Ir) (hydrophilic) for targeted therapy related to breast cancer. The liposomes were prepared with DPPC, cholesterol, and FA-PEG-DSPE using the thin film hydration method (Figure 7A). The size of the blank liposomes, Cs/Ir co-encapsulated conventional liposomes (Lipo/Cs/Ir), and FA-conjugated liposomes (Lipo/Cs/Ir-FA) were around 145, 158, and 174 nm, respectively (Figure 7B). However, the zeta potential of liposomes was not changed significantly after FA conjugation, and all three liposomes exhibited a spherical morphology (Figure 7C). Both of the drugs exhibited higher release profiles at pH 5.0 than at physiological pH (Figure 7D). Furthermore, higher in vitro cell uptake and enhanced apoptosis were observed in FR-positive breast cancer cells (MDA-MB-231 and MCF-7), but not in FR-negative lung cancer cells (A549). In addition, in vivo analysis with MDA-MB-231 xenografts in a mouse tumor model exhibited effective drug delivery response for FA-conjugated liposomes by minimizing the systemic adverse effects and selective targeting of the tumor.

Drug efflux transporters, especially P-glycoprotein (Pgp), restrict the achievements possible with chemotherapy. Nitric oxide (NO) releasing group-conjugated synthetic doxorubicin can overcome resistance by inducing the NO-mediated inhibition of Pgp [138,139,140,141]. Gazzano et al. [26] prepared the first ever folate-conjugated liposomal formulations of nitrooxy-DOX in order to improve active targeting against the Pgp-expressing tumors. Folate-conjugated liposomes were prepared by two different approaches, using the thin film hydration method with distearoylphosphocholine (DSPC), cholesterol, mPEG-DSPE, and FA-PEG-DSPE. One was prepared by adding the FA-PEG-DSPE conjugates with the lipids during liposome preparation, and another was prepared by adding the FA-PEG-DSPE conjugates after the liposomal preparation. Both formulations were compared with respect to their physicochemical properties and in vitro behavior. The prepared nitrooxy-DOX liposomal formulation demonstrated the internalization in an FR-dependent manner and achieved significant anti-tumor efficacy against Pgp-positive and FR-positive cells. In addition, the liposomes delivered the nitrooxy-DOX within the nucleus and triggered mitochondria-dependent apoptosis. This innovative liposomal formulation of multi-target cargo drug, achieving remarkable efficacy against the Pgp-expressing cells, may present significant progress in the treatment of FR/Pgp-positive tumors.

Moghimipour et al. [131] developed folate-targeted liposomes for the delivery of 5-FU to target colon cancer cells. The liposomes were prepared by the thin film hydration method using PC, cholesterol, and FA-PEG-DSPE. The encapsulation efficiency and particle size of the liposomes were about 67% and 114 nm, respectively. Folate-targeted liposomes showed enhanced cellular uptake, with lower IC_50_ (12.02 µM) and higher ROS production (62271.28 fluorescence intensity) compared to free 5-FU (39.81 µM and 2369.55) on CT26 cancer cells. Hemolysis assay revealed that the liposomes were blood biocompatible. Furthermore, 5-FU-encapsulated folate-targeted liposomes exhibited higher tumor inhibition (88.75 mm^3^, tumor volume) compared with free 5-FU (210.00 mm^3^, tumor volume), without any tissue abnormalities, as revealed by histological examination. Thus, it provides a safe and effective chemotherapeutic strategy targeting colon cancer.

Chemotherapy plays an important role in treating Non-Hodgkin lymphoma (NHL), but clinical applications are limited because of the multi-drug resistance and intolerable side-effects [142,143]. FA-modified drug delivery systems have been found to be internalized in B-cell lymphoma in which FRs are overexpressed. Qiu et al. [84] designed and developed a vincristine-loaded folate decorated liposomal system for targeted lymphoma treatment. Vincristine-loaded lipid-polymer hybrid liposomes were prepared by ultrasonication method using poly(lactic-co-glycolic acid (PLGA) and FA-PEG-SA. The encapsulation efficiency of folate-conjugated lipid-polymer hybrid liposomes was more than 90% with particle size of around 174 nm. Vincristine-loaded folate-conjugated liposomes showed higher anti-tumor effect in the murine model bearing lymphoma xenografts and exhibited a targeted effect in vincristine delivery to the B-cell lymphoma cells.

Multifunctional liposomes require complex synthetic processes, which result in low yield, poor reproducibility, and high production cost, thereby presenting difficulties in clinical translation. Ran et al. [57] reported a one-step microfluidic combinatorial technique for synthesizing library of targeted-ligand liposomes with systematically varied properties such as zeta potential, size, targeting ligand, ligand ratio, and ligand density. A cell-penetrating peptide (TAT; CYGRKKRRQRRR) and a targeting ligand (FA) were used to achieve synergistic targeting effect. The liposomes were synthesized by microfluidic hydrodynamic flow focusing method using dimyristoyl glycerophosphocholine (DMPC), cholesterol, dierucoyl glycerophosphoethanolamine (DEPE)-PEG, FA-PEG-DSPE, and DSPE-PEG-TAT and fluorescence-labeled by carbocyanine dyes (DiI or DiR). The liposomes showed enhanced monolayer cellular uptake and 3D tumor spheroid penetration because of the synergistic effect of both the targeting ligands (TAT and FA). In vivo study further verified the enhanced tumor targeting and longer (up to 72 h) tumor retention of the liposomes.

The use of various antitumor agents for cancer treatment is limited due to their low specificity. To overcome this, Monteiro et al. [27] reported the preparation of paclitaxel-loaded pH-sensitive, long-circulating folate-coated liposomes radio-labeled with technetium-99 m (99mTc) for the treatment of solid tumors. The liposomes were prepared using the thin film hydration method with DOPE, CHEMS, DSPE-PEG, DSPE-PEG-diethylenetriaminepentaacetic acid (DTPA), and FA-PEG-DSPE, and they exhibited an encapsulation efficiency of more than 80% and a particle size of around 152 nm. The liposomes were further radio-labeled with ^99m^Tc in order to perform pharmacokinetics and biodistribution studies, the results of which showed high in vitro stability (>90%) and radiochemical purity (>98%). Biodistribution studies and scintigraphic images revealed a significant uptake in liver, kidney, and spleen, indicating these as routes for excretion. ^99m^Tc labeled PTX-loaded folate coated liposomes showed higher uptake than ^99m^Tc-PTX, 8h post-injection and 4 h after administration. Moreover, folate-coated liposomes resulted in a higher tumor-to-muscle ratio than conventional liposomes and free PTX, demonstrating its feasibility as an efficient PTX delivery system to FR-positive tumors

### 3.2. Folate-Targeted Liposomes for Gene Delivery

Nucleic acid therapeutics have emerged as an effective modality for regulating the cellular expression levels of specific genes. They control the expression levels of functional proteins by regulating pathogenic cellular machinery, unlike chemical drugs, which bind to the target proteins for their action. Therefore, nucleic acid therapeutics must penetrate the cell membrane to reach the cytoplasm. Various nucleic acid-based substances have emerged as next-generation therapeutics including small interfering RNA (siRNA), micro RNA (miRNA), antisense oligodeoxynucleotides (asODN), and plasmid DNA (pDNA). However, their utilization has been limited because of relatively weak delivery into the targeted cells due to large size and highly negative charge [144,145,146]. Liposomes have been explored extensively to improve the stability of nucleic acid therapeutics in the bloodstream and to enhance their cellular delivery.

Kabilova et al. [147] developed a novel folate conjugate (FC) built of FA and 1,2-di-O-ditetradecyl-rac-glycerol connected via PEG for the targeted delivery of nucleic acid into FR expressing tumor cells. The liposomes were prepared using 1,26-bis(cholest-5-en-3β-yloxycarbonylamino)-7,11,16,20-tetraazahexacosan tetrahydrochloride (2X3), DOPE and FC with a particle size of around 60 nm, which were non-toxic to the KB-3-1 and HEK 293 cells. The complexes of liposomes with nucleic acids (pEGFP-C2 and siRNA) were synthesized at various nitrogen to phosphate (N/P) ratios for the optimization of cell and liposome interactions. Folate-targeted liposomal delivery of different nucleic acids at low N/P ratios (2/1 and 1/1) showed higher transfection efficiency compared with conventional liposomal formulation. Folate-targeted liposomes at an N/P ratio of 1/1 efficiently accumulated in kidneys and remained there for 24 h, causing downregulation of Pgp expression in tumors. Thus, FC-containing liposomes proved to be effective in the folate-targeted delivery of nucleic acids in xenograft tumors in vivo.

The RNA binding protein human antigen R (HuR) is overexpressed in several human cancers and regulates the expression of various oncoproteins. Furthermore, the overexpression of HuR in the cancer cells has been related with therapy resistance and poor prognosis. Therefore, it was hypothesized that the targeted inhibition of HuR should suppress several oncoproteins in the cancer cells, resulting in a potent anticancer effect. To test this hypothesis, Muralidharan et al. [132] prepared FR-α-targeted liposomes carrying HuR siRNA using FA-PEG-DSPE, 1,2-dioleoyl-3-trimethylammonium-propane chloride salt (DOTAP), and cholesterol with a particle size of 303 nm and a surface charge of +4.3 mV. Serum stability and gel retardation assays revealed that the liposomes protected the rapid degradation of siRNA. The therapeutic efficacy of folate-conjugated liposomes was tested with FR-α-overexpressed human lung cancer cells (H1299) and compared with normal lung fibroblast cells (CCD16), which showed significantly higher uptake and enhanced cytotoxicity against H1299 cells than CCD16 cells. Folate-conjugated liposomes demonstrated efficient internalization in H1299 cells via FR-α-mediated endocytosis. In addition, induced apoptosis and cell-cycle arrest resulted in the significant growth inhibition.

Genome editing based on clustered regularly interspaced short palindromic repeats (CRISPR)-caspase 9 (Cas9) is a highly effective tool for easily generating insertion, deletion, and replacement in the mammalian genome [148,149,150,151]. Thus, CRISPR-Cas9 system holds remarkable potential in repairing or inactivating disease-causing genes. However, the paucity of effective and safe delivery systems impedes its biomedical application. He et al. [133] utilized the FR-targeted liposomes to deliver single guide RNA and CRISPR pDNA co-expressing-Cas9 to target DNA methyltransferase 1 gene (gDNMT1), which is related to ovarian cancer. The liposomes were prepared using DOTAP, cholesterol, FA-PEG-CHEMS, and methoxy-PEG-succinyl cholesterol using the thin layer hydration method. The liposomes bound with gDNMT1 plasmid effectively and carried out the injection of the safe and stable complex. The complex efficiently mutated endogenous DNMT1 in vitro, and then downregulated DNMT1 and expressed the Cas9 endonuclease in vivo. Furthermore, the complex inhibited the tumor growth of paclitaxel-resistant and -sensitive ovarian cancers, thus presenting favorable delivery vectors of the CRISPR-Cas9 technology for the precise genome-editing therapeutics.

Malignant melanoma represents the most threatening form of skin cancer, and its therapy is limited by multidrug resistance and low topical drug concentration. Chen et al. [134] developed folate-conjugated cationic liposomes to deliver hypoxia-inducible factor-1α small interfering RNA (HIF-1α siRNA) for malignant melanoma therapy. HIF-1α siRNA-loaded cationic liposomes were prepared using soybean lecithin S100, cholesterol, and folate/oleic acid-diacylated oligochitosan by the thin film hydration method and subsequently incubated in HIF-1α siRNA. The prepared liposomes exhibited remarkable loading capacity and siRNA protective effect and showed enhanced anti-melanoma activity.

### 3.3. Folate-Targeted Liposomes for Rheumatoid Arthritis

Rheumatoid arthritis is a chronic autoimmune inflammatory disease characterized by synovial joint inflammation and bone tissue destruction [152]. Activated macrophages release inflammatory cytokines, which cause inflammation, bone erosion, joint swelling, and cartilage damage, resulting in swelling, stiffness, pain, and functional impairment [153,154,155]. Low bioavailability, limited selectivity, and high clearance of various important rheumatoid arthritis treatment drugs demand frequent and high dosages to achieve the therapeutic level. However, the use of high doses can increase the risk of systemic side effects. The delivery of these drugs via liposomes increases their therapeutic index by optimized penetration and retention at the administration site, resulting in the prevention of premature degradation and non-targeted tissue toxicity. Furthermore, FA conjugation can further add the advantage of targeted release of these therapeutics. Several folate-targeted liposomes have been explored in recent years for the treatment of rheumatoid arthritis.

To determine whether over-expression of high-affinity FR on the activated macrophages could be beneficial in selectively targeting the imaging agents at the inflammation site in adjuvant-induced arthritic rats, Turk et al. [28] performed folate-targeted imaging via liposomal formulation using ^99m^Tc-EC20 (FA-conjugated ^99m^Tc). Liposomes were prepared with egg PC and cholesterol with 6:4 molar ratio by thin film hydration method. Tissue FR levels were determined using radio-ligand binding assay and found that EC20 was deposited in arthritic diseased rats but not in healthy rats. Increased FR levels and enhanced uptake of EC20 were shown in the spleens and livers of arthritic rats. Furthermore, macrophages obtained from the livers of rats with adjuvant-induced arthritis resulted in significantly enhanced binding capacity for folate-targeted systems compared with macrophages isolated from healthy rats.

Methotrexate is the first line of treatment for rheumatoid arthritis, but many patients are unresponsive to it. To enhance the efficacy and tolerance of methotrexate, Nogueira et al. [135] reported folate conjugation using SP-DS3 peptide linker for rheumatoid arthritis. Methotrexate-loaded folate-conjugated liposomes were prepared by the thin hydration method using DOPE, cholesterol, and N-(carbonyl methoxypolyethylene glycol-2000)-1,2-distearoyl-sn-glycero-3-phosphoethanolamine (DSPE-MPEG) containing FA-peptide (SP-DS3) for targeting and FITC/Alexa Fluor 647 for labeling. Folate-conjugated liposomes showed enhanced internalization in the FR-β-expressed cells compared to the negligible FR-expressed cells. In vivo study with mice bearing arthritis revealed that folate-targeted liposomal formulation strongly accumulated in the joints and displayed superior action by preventing the onset of arthritis in the collagen-induced arthritis model.

**Figure 8:**
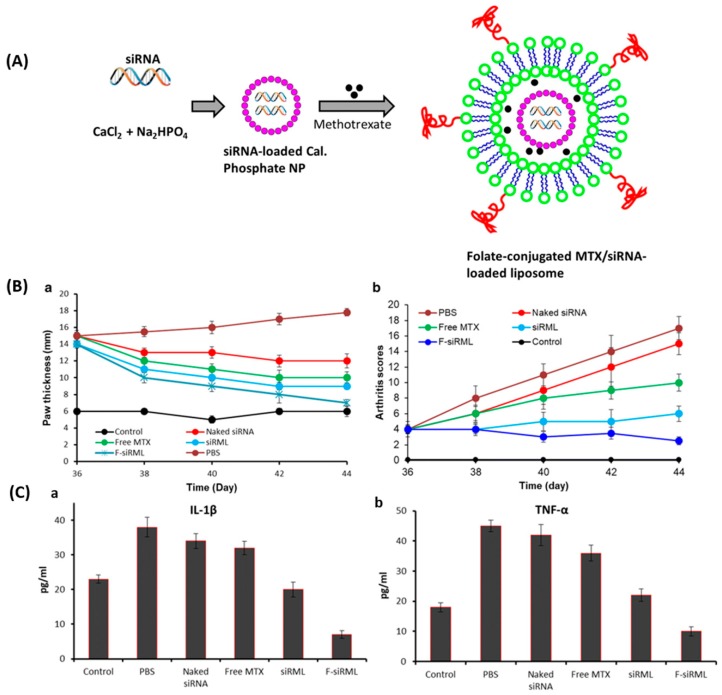
(**A**) Schematic presentation of formation targeted liposomes loaded with CaP/siRNA nanoparticle and methotrexate. (**B**) Therapeutic efficacy of F-siRML and siRML evaluated in arthritic mice. (**a**) Hind paw thickness, (**b**) arthritic scores. (**C**) Analysis of pro-inflammatory cytokines level (**a**) IL-1β and (**b**) TNF-α from serum. All the formulations were injected into arthritic mice, and serums were collected from mice at specified intervals after the administration (day 44). Reprinted with permission from [136]. Copyright 2019, BioMed Central Ltd.

In the pathogenesis of the rheumatoid arthritis, transcription factor NF-kB (nuclear factor kB) plays a crucial role. Since effective rheumatoid arthritis treatment is hindered because of limited specificity of the small-molecules in inflamed joints, Duan et al. [136] developed liposomes encapsulated with the unique combination of methotrexate and siRNA in calcium phosphate (CaP) for the treatment of rheumatoid arthritis. The siRNA/CaP and methotrexate loaded liposomes (Figure 8A) were prepared using DSPC, cholesterol, PEG-DSPE, and FA-PEG-DSPE by the thin film hydration method with a particle size of around 170 nm. Conventional (siRML) and folate-conjugated (F-siRML) systems showed a sustained release profile for methotrexate and siRNA up to 80 h. The therapeutic efficacy of the formulations was evaluated in terms of arthritic scores and paw thickness (Figure 8B). F-siRML did not show an increase in the arthritis score, and the same joint score was maintained throughout the whole study, while paw thickness was also significantly suppressed, indicating its remarkable therapeutic efficacy. The folate-targeted system showed significant suppression of arthritis progression in the mouse model and effectively blocked NF-kB signaling pathway, as well as showing a reduction in pro-inflammatory cytokine (IL-1β and TNF-α) expression (Figure 8C), thereby providing a promising approach for the treatment of rheumatoid arthritis.

Activated macrophages highly upregulate the FR-β and also play a crucial role in development of inflammatory diseases, thus providing a mechanism to selectively deliver therapeutic agents by utilizing these FRs as a target. Scott Poh et al. [137] prepared the betamethasone-loaded folate-conjugated PEG-coated liposomes to target the FRs related to inflammatory diseases such as rheumatoid arthritis, atherosclerosis, and psoriasis. The liposomes were prepared by the thin film hydration method using the lipid compositions, DSPC, cholesterol, PEG-DSPE, and FA-PEG-DSPE with betamethasone loading and fluorescence-labeled by carbocyanine dyes (DiD). The prepared folate-conjugated liposomes specifically accumulated in FR positive cells at the inflammation sites to selectively deliver therapeutic and imaging agents to sites in the mouse models of atherosclerosis as well as colitis.

## 4. Concluding Remarks and Future Directions

In this review, various characteristics of folate-conjugated liposomes, fabrication strategies, and their applicability in biomedical applications have been discussed. Liposomes mainly consist of phospholipids, cholesterol, and sometimes targeted peptides, and FA is generally attached to these prior to liposomal formation. This method has been proved to be highly effective, achieving a variety of successful folate-targeted liposomal deliveries [156,157,158] and offering many advantages. The advantages and disadvantages of folate-conjugated phospholipids, cholesterol, and peptides are summarized in Table 2. FA is more tumor selective, easier to conjugate, less immunogenic, smaller in size, and cheaper than most of the other targeting ligands under investigation. Since the specificity for the pathogenic cells is very high, targeting ligands such as folate-targeted liposomes can not only promote enhanced drug uptake by the diseased cells but also profoundly reduce the collateral normal tissue toxicity. Due to its targeting ability, not only cancer, but also a variety of inflammatory diseases can be treated, so it may eventually become more diverse and applicable than other targeting ligands.

Despite several benefits, development of FR-targeted therapies also presents some challenges. First, since relatively few molecules can be transported into the cells via most endocytic pathways, the targeted drugs must be efficiently effective at low concentration. Secondly, many FR-targeted delivery systems have demonstrated the activity only in vitro, which alone could not provide the precise results. Therefore, detailed in vivo examinations and clinical trials are needed in order to realize their applicability in patients. Also, some of the liposome-based anticancer drugs have been reported to be recognized as foreign materials by immune system resulting in adverse immune phenomena such as complement (C) activation, which is a major contributing factor to C activation-related pseudoallergy (CARPA) (hypersensitivity syndrome) [159]. CARPA may become a recurrent safety issue in the near future, and its prevention is necessary at early stages of research and development.

Since the folate-conjugated liposomes proved to be highly effective in various biomedical applications, cleavable folate-conjugated liposomes have rarely been explored. Therefore, folate-conjugated liposomes containing cleavable bonds such as disulfide bonds [121,160], vinyl bonds [161], and peptide bonds [162,163] could also be explored in the near future in order to obtain rapid release after reaching the tumor site. Also, only a few of the folate-conjugated phospholipids, such as FA-PEG-DSPE, FA-PEG-DPPE, and FA-PEG-SA, have been used for the fabrication of FR-targeted liposomes. The applicability of other phospholipids conjugated with FA should also be explored. Moreover, FITC-labeled FA derivative could also be explored for the fabrication of liposomes in order to gain the benefits of diagnosis and targeted therapy. Last, but most important, is the need for an optimized laboratory process to improve the cost-effectiveness and feasibility of the industrial-scale production of folate-targeted liposomes. The above-mentioned problems need to be addressed and further research is essential to identify the clinical applicability of the folate-targeted liposome-based drug delivery system.

## Figures and Tables

**Table 1:** Liposomal-based systems; lipid composition, formulation method, pharmaceutical agent administered and their applications.

Pharmaceutical Agent	Lipids Composition	Liposomes Formulation Method	Application	Ref.
Paclitaxel and ^99m^Tc	DOPE, CHEMS, DSPE-PEG, FA-PEG-DSPE, and DSPE-PEG-DTPA	Film hydration method	Anticancer and imaging (In vitro MDA-MB-231 cells)	[27]
^99m^Tc-EC20 (FA-conjugated ^99m^Tc)	Egg PC and cholesterol	Film hydration method	Rheumatoid arthritis and imaging (In vivo female Lewis rats)	[28]
Dil (carbocyanine dye)	DMPC, cholesterol, dihexadecyl phosphate, DSPE-PEG, and FA-PEG-DSPE	Microfluidic hydrodynamic flow focusing method	Imaging (In vitro RAW 264.7, SKOV3, and MCF-7 cells)	[53]
No drug used	DMPC, cholesterol, dihexadecyl phosphate, DMPE-PEG, and FA-PEG-DSPE	Microfluidic hydrodynamic flow focusing method	Anticancer	[54]
DiI/DiR (carbocyanine dye) and TAT peptide (CYGRKKRRQRRR)	DMPC, cholesterol, DEPE-PEG, FA-PEG-DSPE, and DSPE-PEG-TAT	Microfluidic hydrodynamic flow focusing method	Anticancer and imaging (In vitro RAW 264.7, SKOV3, and MCF-7 cells and In vivo nude Balb/C mice)	[57]
Paclitaxel	SPC, cholesterol, DSPE-PEG-OMe, DSPE-PEG-Hz-FA, and DSPE-PEG-dNP2	Film hydration method	Anticancer (In vitro C6 cells)	[81]
Vincristine	PLGA, FA-PEG-SA	Ultrasonication method	Anticancer (In vitro Raji, HUVEC, and A20 cells)	[84]
DOX and imatinib	DOPE, HSPC, CHEMS, cholesterol, mPEG_2000_-Hz-VES, and FA-PEG-CHEMS	Film hydration method	Anticancer (In vitro MCF-7 and MCF-7/ADR cells)	[97]
Celastrol and irinotecan	DPPC, cholesterol, and FA-PEG-DSPE	Film hydration method	Anticancer (In vitro MCF-7, MDA-MB-231, and A549 cells)	[122]
5-FU	DPPC, cholesterol, and FA-PEG-DSPE	Film hydration method	Anticancer (In vitro HT-29, Caco-2, CT26, HeLa, and MCF-7 cells)	[130]
Paclitaxel and imatinib	DSPC, cholesterol, and FA-PEG-DSPE	Film hydration method	Anticancer (In vitro MCF7 and PC-3 cells)	[129]
5-FU	PC, cholesterol, and FA-PEG-DSPE	Film hydration method	Anticancer (In vitro CT26 cells)	[131]
DNA or siRNA	DOTAP, cholesterol, and FA-PEG-DSPE	Film hydration method	Anticancer (In vitro H1299 and CCD16 cells)	[132]
Single-guide RNA and CRISPR plasmid DNA co-expressing-Cas9	DOTAP, cholesterol, FA-PEG-CHEMS, and methoxy-PEG-succinyl cholesterol	Film hydration method	Anticancer (In vitro gDNMT1 and in vivo Cas9 endonuclease)	[133]
HIF-1α siRNA	Soybean lecithin S100, cholesterol, and folate/oleic acid-diacylated oligochitosan	Film hydration method	Anticancer (In vitro Hypoxia-induced a375 cells)	[134]
Methotrexate	DOPE, cholesterol, DSPE-MPEG, and FA-peptide (SP-DS3)	Film hydration method	Rheumatoid arthritis (In vitro THP-1 macrophages and Jurkat T cells and in vivo mice)	[135]
Methotrexate and siRNA	DSPC, cholesterol, PEG-DSPE, and FA-PEG-DSPE	Film hydration method	Rheumatoid arthritis (In vitro Raw 264.7 cells and in vivo mice)	[136]
Betamethasone and DiD (carbocyanine dyes)	DSPC, cholesterol, PEG-DSPE, and FA-PEG-DSPE	Film hydration method	Imaging, rheumatoid arthritis, atherosclerosis, and psoriasis (In vitro RAW 264.7 cells and in vivo mice)	[137]

Abbreviations: 5-FU: 5-fluorouracil; CHEMS: 3β-hydroxy-5-cholestene-3-hemisuccinate; DiD: 1,1′-dioctadecyl-3,3,3′,3′-tetramethylindodicarbocyanine, 4-chlorobenzenesulfonate salt; Dil: 1,1′-dioctadecyl-3,3,3′,3′-tetramethylindocarbocyanine perchlorate; DiR: 1,1′-dioctadecyl-3,3,3′,3′-tetramethylindotricarbocyanine iodide, DMPC: dimyristoyl glycerophosphocholine; DMPE: dimyristoyl glycerophosphoethanolamine; dNP2: KIKKVKKKGRKKIKKVKKKGRK-cys (peptide); DOPE: 1,2-dioleoyl-sn-glycero-3-phosphoethanolamine; DOTAP: 1,2-dioleoyl-3-trimethylammonium-propane chloride salt; DPPC: dipalmitoylphosphatidylcholine; DSPC: distearoylphosphocholine; DSPE: distearoylphosphatidylethanolamine; DTPA: diethylenetriaminepentaacetic acid; FA: folic acid; HSPC: hydrogenated soybean phosphatidylcholine; Hz: hydrazine; mPEG_2000_-Hz-VES: cleavable d-α-tocopheryl polyethylene glycol succinate (TPGS) analogue; PC: phosphatidylcholine; PEG: poly(ethylene glycol); PLGA: poly(lactic-co-glycolic acid); SA: stearylamine; SPC: soybean phosphatidylcholine; TAT: CYGRKKRRQRRR (peptide).

**Table 2:** Overview of some commonly used folate-conjugated analogues of uncleavable/cleavable phospholipids, cholesterols, and peptides along with their advantages and disadvantages.

Folate-Conjugated Derivatives	Commonly Used Analogues	Advantages	Disadvantages
Uncleavable folate-conjugated phospholipids	FA-PEG-DSPE, FA-PEG-DPPE, and FA-PEG-SA	Excellent specificity towards FRs Required in small amount (incorporation of 0.1 mol% is sufficient) Easy synthetic procedure	Uncleavable under acidic tumor environment
Acid-cleavable folate-conjugated phospholipids	FA-Hz-PEG-DSPE	Stable under physiological environment and rapidly dissociate in acidic environment to provide triggered release of therapeutics Excellent specificity towards FRs. Required in small amount (incorporation of 0.1 mol% is sufficient)	Not cost effective
Folate-conjugated cholesterol	FA-PEG-CHEMS	Restricts the trans to gauche conformational change Stabilizes the lipid membranes against temperature changes, thus provides the stability to the liposomes Excellent specificity towards FRs.	Not cost effective
Folate-conjugated Proteins	SP-DS1, SP-DS2, and SP-DS3	Deep insertion prevents its dissociation from liposome after the intravenous injection α-helical neck section of the SP-D exhibits an affinity for the phospholipids which might stimulate the binding of SP-D with phospholipids of liposomes	Long synthetic procedure Not cost effective
